# Low tumour cell proliferation at the invasive margin is associated with a poor prognosis in Dukes' stage B colorectal cancers

**DOI:** 10.1038/sj.bjc.6690091

**Published:** 1999-02

**Authors:** R Palmqvist, P Sellberg, Å Öberg, B Tavelin, J N Rutegård, R Stenling

**Affiliations:** Department of Pathology, Umeå University, Umeå, Sweden; Department of Surgery, Umeå University, Umeå, Sweden; Department of Oncology, Umeå University, Umeå, Sweden; Department of Surgery, District Hospital of Örnskoldsvik, Örnsköldsvik, Sweden

**Keywords:** cell proliferation, Ki-67, heterogeneity, invasion, colorectal carcinoma

## Abstract

The conflicting results about the prognostic impact of tumour cell proliferation in colorectal cancer might be explained by the heterogeneity observed within these tumours. We have investigated whether a systematic spatial heterogeneity exists between different compartments, and whether the presence of such a systematic heterogeneity has any impact on survival. Fifty-six Dukes' stage B colorectal cancers were carefully morphometrically quantified with respect to the immunohistochemical expression of the proliferative marker Ki-67 at both the luminal border and the invasive margin. The proliferative activity was significantly higher at the luminal border compared with the invasive margin (*P* < 0.001), although the two compartments were also significantly correlated with each other. Tumours with low proliferation at the invasive margin had a significantly poorer prognosis both in univariate (*P* = 0.014) and in multivariate survival analyses (*P* = 0.042). We conclude that Dukes' B colorectal cancers exhibit a systematic spatial heterogeneity with respect to proliferation, and tumours with low proliferation at the invasive margin had a poor prognosis. The present data independently confirm recent results from the authors, and provide new insights into the understanding of tumour cell proliferation in colorectal cancer. © 1999 Cancer Research Campaign


*British Journal of Cancer* (1999) **79**(3/4), 577–581
© 1999 Cancer Research Campaign
Article no. bjoc.1998.0091

### Low tumour cell proliferation at the invasive margin is associated with a poor prognosis in Dukes' stage B colorectal cancers

**R Palmqvist[1], P Sellberg[1], Å Öberg[2], B Tavelin[3], JN Rutegård[2,4] and R Stenling[1]**

Department of [1]Pathology, [2]Surgery and [3]Oncology, Umeå University, Umeå, Sweden; [4]Department of Surgery, District Hospital of Örnskoldsvik, Örnsköldsvik, Sweden

**Summary** The conflicting results about the prognostic impact of tumour cell proliferation in colorectal cancer might be explained by the heterogeneity observed within these tumours. We have investigated whether a systematic spatial heterogeneity exists between different compartments, and whether the presence of such a systematic heterogeneity has any impact on survival. Fifty-six Dukes' stage B colorectal cancers were carefully morphometrically quantified with respect to the immunohistochemical expression of the proliferative marker Ki-67 at both the luminal border and the invasive margin. The proliferative activity was significantly higher at the luminal border compared with the invasive margin ($P < 0.001$), although the two compartments were also significantly correlated with each other. Tumours with low proliferation at the invasive margin had a significantly poorer prognosis both in univariate ($P = 0.014$) and in multivariate survival analyses ($P = 0.042$). We conclude that Dukes' B colorectal cancers exhibit a systematic spatial heterogeneity with respect to proliferation, and tumours with low proliferation at the invasive margin had a poor prognosis. The present data independently confirm recent results from the authors, and provide new insights into the understanding of tumour cell proliferation in colorectal cancer.

**Keywords:** cell proliferation; Ki-67; heterogeneity; invasion; colorectal carcinoma

Classically, neoplasia has been considered to be primarily a disturbance in the regulation of proliferation, or at least deregulation has been proposed to be essential for cancer development (Hartwell and Kastan, 1994). It is, therefore, not surprising that a high proliferative activity is usually correlated with a more aggressive behaviour and a poorer prognosis in many malignancies. In some malignancies, tumour cell proliferation has been reported to be a strong independent prognostic factor, e.g. in breast cancer, bladder cancer and malignant lymphomas (Porter-Jordan and Lippman, 1994; Mulder et al, 1992; Hall et al, 1988).

In colorectal cancer, however, the associations between tumour cell proliferation and prognosis are more difficult to interpret. Conflicting results have been reported and the differences between reports are most probably independent of the choice of proliferation marker. S-phase fraction, proliferative cell nuclear antigen (PCNA), Ki-67 and bromo- or iododeoxyuridine (BrdUrd/IdUrd) have all been regarded as prognostic indicators in colorectal cancer. Although some reports suggest a correlation between rapid tumour cell proliferation and poor prognosis (Witzig et al, 1991; Pietra et al, 1996), most reports find no support for proliferation as a prognostic factor (Kubota et al, 1992; Al-Sheneber et al, 1993; Rew, 1993; Zarbo et al, 1997), and some indicate a better prognosis for rapidly proliferating colorectal cancer (Neoptolemos et al, 1995; Paradiso et al, 1996). These results have been very confusing and have given rise to discussions and studies of strictly technical topics (Bauer et al, 1993).

Colorectal cancers are known to be heterogeneous with respect to several parameters, including tumour cell proliferation (Jass et al, 1986; Shepherd et al, 1988; Koha et al, 1990; Lindmark et al, 1991), and such heterogeneity could partly explain the conflicting results. Systematic heterogeneity was found in human colorectal cancers after in vivo incorporation of IdUrd, with the luminal border showing a higher proliferation than the invasive margin (Palmqvist et al, 1998). In addition, we reported a correlation between slowly proliferating tumours (low fraction of proliferative cells and/or long potential doubling time) and a poor prognosis. This correlation was observed for both the luminal border and the invasive margin. Our results suggested that one must control for heterogeneity when measuring the proliferative activity within colorectal cancers, and that sampling from defined compartments is desirable.

The biological explanation for the systematic heterogeneity is not known. Several faecal factors, such as secondary bile acids and short chain fatty acids, are known to have an effect on proliferation in colonic cells (Mullan et al, 1990; Butler et al, 1992), and therefore these factors could be responsible for the systematic differences between separate compartments in colorectal cancer. If this hypothesis is correct, the proliferative activity is expected to decrease as the diffusion distance for faecal factors increases.

The aims of this study were to investigate the prognostic impact of tumour cell proliferation using an endogenous marker for cell proliferation, when the systematic heterogeneity within the colorectal cancers was taken into consideration. We also evaluated whether the proliferative activity at the invasive margin was dependent on the depth of invasion in the bowel wall and on other clinicopathological parameters.

*Received 19 January 1998*
*Revised 6 May 1998*
*Accepted 16 June 1998*

*Correspondence to:* R Stenling, Umeå University, Department of Pathology, SE-901 87 Umeå, Sweden

### MATERIALS AND METHODS

### Patients

Fifty-six patients with Dukes' stage B colorectal cancer (Dukes, 1932), who underwent surgery at the University Hospital of Umeå, were included in this retrospective study. The patients had undergone surgery during the period 1987–92. Out of the 56 colorectal cancer patients, 47 did not receive adjuvant chemotherapy, and underwent potential curative surgery treatment and were regarded as cured if both the surgeon (ÅÖ) and the pathologist (RS) believed that all tumour tissue had been removed. Only these 47 patients were included in the survival analyses. Out of the 56 patients, 31 (55%) were men and 25 (45%) were women. Seventeen tumours (30%) were located in the right colon (defined as caecum, colon ascendens and colon transversum), 24 (43%) in the left colon (colon descendens and colon sigmoideum) and 15 (27%) in the rectum. The median age of the patients was 72.0 (range 39–89), with five patients younger than 60 years of age, 17 between 60 and 69 years, 21 between 70 and 79 years, and 13 patients 80 years of age or older. The follow-up time of the surviving patients ranged from 46 to 106 months (median 67.0). To verify the accuracy of corrected survival used in this study, curves of corrected survival for the entire material were compared with relative survival data, i.e. death rates of the colorectal cancer group compared with death rates of a corresponding normal population. These two survival curves showed good agreement.

### Histology

Each colorectal cancer was classified by the pathologist (RS) according to grade (high, low), growth pattern at the invasive margin (pushing or infiltrating), the degree of lymphocytic infiltration at the invasive margin (low or high), tumour type (mucinous or not mucinous) and the presence of vascular invasion (yes or no). In addition, the maximum depth of tumour invasion observed (in four sections) for each tumour was measured with a millimetre ruler.

### Immunohistochemical procedures

Each tumour was systematically sampled by dividing the tumour into two central and two peripheral regions. From each region, a tumour sample cut perpendicular to the mucosal surface was collected and fixed overnight in phosphate-buffered neutral 10% formalin and subsequently embedded in paraffin. A 4-μm thick section was cut from each paraffin block, including the entire tumour, i.e. from the intestinal lumen all the way to the submucosa, muscular layer or to the peripheral fat. The sections (four from each tumour) were left to dry overnight in 37°C, followed the next morning by 30 min in 56°C. After dewaxing and rehydration, the slides were microwaved (750 W) in citrate buffer (pH 6.0) for 4 × 5 min, followed by a staining procedure in an automated immunostainer (Ventana ES, Ventana, Tucson, AZ, USA) to obtain the most constant conditions (Grogan, 1992; Nichols et al, 1996). A monoclonal Ki-67 antibody (A0047, Dako, Denmark) was used at a dilution of 1:75, followed by antibody visualization according to the Ventana-program. Subsequently, the slides were manually counterstained with Mayer's haematoxylin for 1.5 min. The Ventana ES is constrained to perform all incubations at 40°C slide reaction temperature. From each tissue block, adjacent samples were also taken for routine histological evaluations. In all daily staining procedures, tissue samples from normal colon epithelium were included as a positive control.

### Morphometrical analysis

The methods used have recently been described in detail by Palmqvist et al (1998). Briefly, one immunohistochemically stained section from each of the four tissue samples (collected as described above) was used for morphometrical analysis. A Zeiss microscope (40 × objective magnification) equipped with an eyepiece squared graticule was used to define the proper size of the counting frame in each of the tumours (Gundersen et al, 1988). From each tumour, counting frames from about 40 fields of vision were measured in a systematic random fashion, i.e. the first field was selected at random whereas subsequent fields were sampled systematically by adjusting the distance between individual fields of vision so that they were roughly proportional to the overall area in question. Two tumour compartments were evaluated in each colorectal cancer section. One was represented by the most superficial fourth (corresponding to the luminal tumour part), whereas the other was represented by the deepest fourth (corresponding to the invasive margin). The two tumour compartments were analysed separately without knowledge of the results from earlier morphometrical evaluation.

Two different unbiased counting frames were used for counting nuclei, i.e. one for positive and the other for negative nuclei (Gundersen, 1977). In each field of vision, both the number of positive and negative nuclei were counted in each of the defined counting frames, respectively, and subsequently the two-dimensional numerical density was calculated for both positive and negative nuclei. The labelling index (LI) was calculated as the two-dimensional numerical density for positive nuclei divided by the total two-dimensional numerical density for both positive and negative nuclei.

To investigate the variability of the morphometrical measurements, we performed repetitive measurements of LI, as described above, in four randomly chosen tumours. The coefficient of error (CE) for differences between paired measurements at the luminal border was 0.059 for intraobserver variability and 0.087 for interobserver variability. Regarding the measurements at the invasive margin, the CE was 0.049 for intraobserver variability and 0.085 for interobserver variability.

### Statistics

Spearmann's correlation coefficient (rho) and Wilcoxon matched-pairs signed-ranks test were used to compare sets of continuous parameters measured on the same tumour. Differences between groups were examined using the Kruskal–Wallis test. In a univariate survival analysis, Kaplan–Meier's method was used to estimate corrected survival, and comparison between groups was performed with the log-rank test. Corrected survival analyses measure the proportion of deaths resulting from cancer. The time measured from operation to death is recorded as the survival time, in which death with known locoregional or distant metastases was processed as an event. If no event occurred, the patient was censored at the time of last clinical follow up or death from other causes.

To evaluate the simultaneous effect of different factors on survival, the Cox proportional hazard model was used. The hypothesis that the coefficients of each variable were equal to 0 was tested using the global chi-squared test. A *P*-value less than 0.05 was required for statistical significance and two-sided tests were performed for all analyses. Statistical analyses were performed using SPSS version 7.5 (SPSS, IL, USA).

© Cancer Research Campaign 1999

**Table 1** Results of Cox proportional hazard model in 47 colorectal Dukes, B cancers

| Variable | Relative risk ($e^{\beta}$) | 95% Confidence interval | *P*-value |
|---|---|---|---|
| Gender | | | |
| Man | 1.00 | | |
| Woman | 0.37 | 0.06–2.11 | 0.26 |
| Age[a] | 1.06 | 0.96–1.18 | 0.21 |
| Tumour type | | | |
| Mucinous | 1.00 | | |
| Not mucinous | 0.47 | 0.05–4.27 | 0.50 |
| Growth pattern | | | |
| Pushing | 1.00 | | |
| Infiltrating | 4.41 | 0.93–21.00 | 0.062 |
| Vascular invasion | | | |
| Present | 1.00 | | |
| Absent | 1.61 | 0.30–8.47 | 0.58 |
| Grade | | | |
| High | 1.00 | | |
| Low | 1.21 | 0.28–5.19 | 0.80 |
| Lymphocytic reaction | | | |
| Low | 1.00 | | |
| High | 0.62 | 0.15–2.58 | 0.51 |
| Depth of invasion[a] | 1.14 | 0.92–1.41 | 0.25 |
| Ki-67 luminal border | | | |
| Above lowest quartile | 1.00 | | |
| Lowest quartile | 0.22 | 0.03–1.81 | 0.16 |
| Ki-67 invasive margin | | | |
| Above lowest quartile | 1.00 | | |
| Lowest quartile | 12.11 | 1.10–133 | 0.042 |

[a]Continuous variables.

### RESULTS

Among the 56 colorectal cancers, we found a mean value of 43.7% for Ki-67 LI at the luminal border compared with 36.8% at the invasive margin. The latter value was significantly lower in a paired test ($P < 0.001$). In only ten cases was LI higher at the invasive margin compared with the luminal border. Nevertheless, LI at the luminal border and the invasive margin were correlated (rho = 0.829; $P < 0.001$). In contrast, LI at the invasive margin was not correlated with the infiltrative depth of the tumour within the bowel wall (rho = –0.032; $P = 0.814$).

There was no difference in proliferative activity in relation to tumour site ($P = 0.969$). The mean values from the luminal border in different sites were 40.3 ± 3.2 (s.e.m.) (range 20.3–66.8), 45.9 ± 3.6 (13.2–77.6) and 43.9 ± 3.3 (21.9–69.4) for right colonic, left colonic and rectal tumours respectively. For measurements at the invasive margin, the mean values were 37.1 ± 3.0 (19.1–58.9), 36.8 ± 2.9 (12.7–63.6) and 36.6 ± 3.0 (15.3–57.1) respectively.

Out of the 47 patients included in the survival analyses, 29.8% died from their cancer disease, whereas 70.2% was censored. Using the lowest quartile as a cut-off level (LI = 27.4%) in a univariate analysis, a significantly poorer survival ($P = 0.0139$) was detected for patients with low LI at the invasive margin (Figure 1). In contrast, no such impact on survival was recorded for LI at the luminal border ($P = 0.705$). If, instead, median values were used as cut-off levels, low LIs at both luminal border and

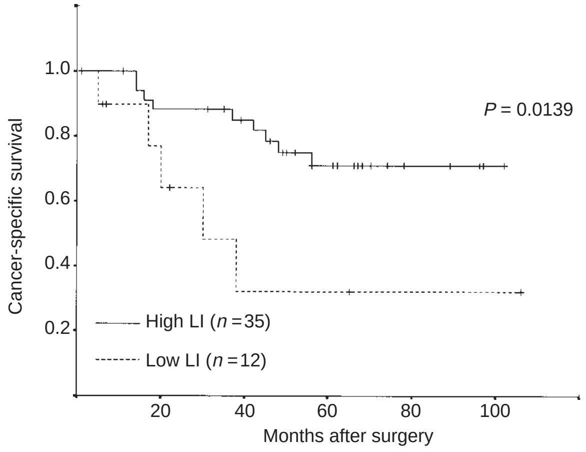

**Figure 1** Kaplan–Meier surviving curve showing impaired survival for patients with low (lowest quartile used as cut-off level) Ki-67 labelling indices (LIs)

invasive margin were significantly associated with poorer prognosis ($P = 0.0167$ and $P = 0.0439$ respectively).

The clinicopathological parameters age, gender, grade, vascular invasion, growth pattern at invasive margin, tumour type, depth of tumour invasion, degree of lymphocytic infiltration and LI at the invasive margin were put into a multiple Cox regression model for a better estimation of the prognostic significance of these variables. The global chi-squared test was significant ($P = 0.0072$) indicating that our model was adequate, but one should nevertheless be aware of the small number of events in the present study and the consequently poor statistical power. Only LI at the invasive margin, dichotomized using the lowest quartile as the cut-off level, turned out to be a significant prognostic marker (relative risk (RR) = 12.1; $P = 0.042$). Although not statistically significant, a borderline value was observed for the growth pattern at the invasive margin (RR = 4.41; $P = 0.062$; Table 1).

### DISCUSSION

Although the approach used in the present study is similar to that of our recent study (Palmqvist et al, 1998), it differs both with respect to the proliferation marker and the patient material studied. This was carried out to reduce the random risk of investigating a non-representative population. We retrospectively collected a group of Dukes' stage B colorectal cancers, which most probably have the greatest benefit by an improvement of prognostic tools. We have used a well-established morphometrical method to measure the immunohistochemical outcome of Ki-67 (Weibel, 1979; Gundersen, 1986; Gundersen et al, 1988). Only one exception from the theoretically most desirable design was made, i.e. in the first sampling step we have used a strictly systematic sampling in contrast to a sampling scheme with a random start followed by a systematic sampling. This systematic procedure was chosen because it is more feasible in clinical practice, and we have recently shown that it causes only a minor sampling error (Palmqvist et al, 1998). When analysing the luminal border and the invasive margin, a strict definition was used, i.e. the most superficial and deepest fourth of the tumour depth was evaluated respectively. This definition was chosen for both simplicity and reproducibility.

© Cancer Research Campaign 1999

In the present study, the proliferative activity was found to be systematically higher at the luminal border compared with the invasive margin in colorectal cancers. Quirke et al (1985) considered the idea of systematic heterogeneity already in 1985, but did not detect a significant difference between superficial and deep compartments in colorectal cancers. This was probably due to the use of flow cytometry instead of immunohistochemistry. With flow cytometry, it is difficult both to define separate tumour compartments and to avoid contamination of the samples with non-tumour cells, but these problems are, however, technically possible to solve using microdissection and cytokeratin-based gating. Our data are supported by reports after in vitro labelling with BrdUrd (Taniyama et al, 1993) and by the distributions of Ki-67-positive tumour and endothelial cells (Vermeulen et al, 1995) in colorectal cancers. Furthermore, the same proliferative pattern as described in this study has recently been shown in gastric carcinomas (Ramires et al, 1997). The striking systematic heterogeneity with higher proliferative activity at the luminal border compared with the invasive margin emphasize the importance of measuring well-defined tumour areas when evaluating parameters associated with proliferation in polarized tumours such as colorectal cancer.

Factors such as secondary bile acids (mainly lithocholic acids), other steroid-derived compounds, short-chain fatty acids and the direct influence from the bacterial content are known to affect the proliferation of colorectal cells (Mullan et al, 1990). However, we found no correlation between the depth of tumour invasion and the proliferative activity at the invasive margin. This result is not consistent with the hypothesis that increased proliferation of the cells at the luminal border is induced by the luminal content. If this were true, proliferation rates should decrease as the distance from the luminal contents increases. Other luminal occurrences can, of course, be involved, e.g. the preoperative colon-cleaning preparations, such as laxatives and enemas, have been shown to induce an increased mucosal proliferative activity (Lehy et al, 1984). Not only these luminal occurrences but also local non-specific regenerative factors from the ulcerative process at the luminal border may increase the proliferation in this compartment.

The significant association between low tumour cell proliferation at the invasive margin and poor prognosis in Dukes' B colorectal cancer, which were recorded in both uni- and multivariate analyses, provides new insights about the influence of tumour cell proliferation on biological behaviour, and might also separate colorectal cancer from other types of cancers, e.g. breast cancers and malignant lymphomas. Nevertheless, there are reports which, directly or indirectly, support this new view of the relation between proliferation and prognosis in colorectal cancer. From two studies on the invasive margin in colorectal cancer, Taniyama et al (1993, 1996) report that low tumour cell proliferative activity is correlated with areas of low differentiation, and that decreased proliferation in diploid tumours is correlated with increased numbers of lymph node metastases. Another circumstantial support is that the proliferation rate decreases when the colorectal cancer stage increases (Kubota et al, 1992; Roncucci et al, 1992). Furthermore, low PCNA indices in a group of patients with advanced colorectal cancers given chemotherapy have been shown to be correlated with a poorer prognosis (Paradiso et al, 1996). There are, however, other results, mainly from flow cytometrical studies of S-phase fraction, indicating a better prognosis for patients with low tumour cell proliferation (Harlow et al, 1991; Witzig et al, 1991).

The present study does not separate tumour from colon and rectum and considers only Dukes' stage B colorectal cancers. Speculatively, if low proliferative activity at the invasive margin merely is a secondary phenomenon to the metastasizing process and, therefore, mainly would be a measurement of the likelihood of having metastases, it would not be surprising to find patients with highly proliferative tumours and already established distant spread (Dukes' D) to have a very poor prognosis. Furthermore, the relation between proliferation and apoptosis is another interesting track when trying to understand why patients with tumours showing high proliferative activity have a favourable outcome. Tumour cells with high proliferative activity might be those having an imbalance in 'proapoptotic' and 'antiapoptotic' signals making them more vulnerable to apoptotic death, whereas tumour cells with low proliferation might be those being more in balance and therefore will survive (Evan, 1997). Further studies are needed to clarify the role of the proliferative activity in other Dukes' stages and in relation to apoptosis and topography.

To summarize, we conclude that the proliferative activity is higher at the luminal border compared with the invasive margin in the main part of colorectal cancers and, moreover, that a low proliferative activity is correlated with a poorer prognosis in Dukes' B colorectal cancer. This new insight into tumour cell proliferation might have impact on future treatment of colorectal cancer.

### ACKNOWLEDGEMENTS

This report was supported by grants from Swedish Cancer Society project no. 2520-B96-08XAC, from the Lion's Cancer Research Foundation, Umeå, Sweden and from the Medical Faculty of Umeå University. Mrs Kerstin Näslund has contributed to this paper by skilful technical assistance.

### REFERENCES

Al-Sheneber IF, Shibata HR, Sampalis J and Jothy S (1993) Prognostic significance of proliferating cell nuclear antigen expression in colorectal cancer. *Cancer* **71**: 1954–1959

Bauer KD, Bagwell CB, Giaretti W, Melamed M, Zarbo RJ, Witzig TE and Rabinovitch PS (1993) Consensus review of the clinical utility of DNA flow cytometry in colorectal cancer. *Cytometry* **14**: 486–491

Butler RN, Bruhn B, Pascoe V, Fettman MJ and Roberts Thomson IC (1992) Regional factors affecting proliferation in the large intestine of the rat. *Proc Soc Exp Biol Med* **200**: 133–137

Dukes CE (1932) The classification of cancer of the rectum. *J Pathol Bacteriol* **35**: 323–332

Evan G (1997) Cancer – a matter of life and cell death. *Int J Cancer* **71**: 709–711

Grogan TM (1992) Automated immunohistochemical analysis. *Am J Clin Pathol* **98**: S35–S38

Gundersen HJG (1977) Notes on the estimation of the numerical density of arbitrary profiles: the edge effect. *J Microsc* **111**: 219–223

Gundersen HJG (1986) Stereology of arbitrary particles. A review of unbiased number and size estimators and the presentation of some new ones, in memory of William R Thompson. *J Microsc* **143**: 3–45

Gundersen HJG, Bendtsen TF, Korbo L, Marcussen N, Moller A, Nielsen K, Nyengaard JR, Pakkenberg B, Sorensen FB, Vesterby A and West MJ (1988) Some new, simple and efficient stereological methods and their use in pathological research and diagnosis. *APMIS* **96**: 379–394

Hall PA, Richards MA, Gregory WM, D'Ardenne AJ, Lister TA and Stansfeld AG (1988) The prognostic value of Ki67 immunostaining in non-Hodgkin's lymphoma. *J Pathol* **154**: 223–235

Harlow SP, Eriksen BL, Poggensee L, Chmiel JS, Scarpelli DG, Murad T and Bauer KD (1991) Prognostic implications of proliferative activity and DNA aneuploidy in Astler-Coller Dukes stage C colonic adenocarcinomas. *Cancer Res* **51**: 2403–2409

Hartwell LH and Kastan MB (1994) Cell cycle control and cancer. *Science* **266**: 1821–1828

© Cancer Research Campaign 1999

Jass JR, Atkin WS, Cuzick J, Bussey HJ, Morson BC, Northover JM and Todd IP (1986) The grading of rectal cancer: historical perspectives and a multivariate analysis of 447 cases. *Histopathology* **10**: 437–459

Koha M, Caspersson TO, Wikström B and Brismar B (1990) Heterogeneity of DNA distribution pattern in colorectal carcinoma. A microspectrophotometric study of fine needle aspirates. *Anal Quant Cytol Histol* **12**: 348–351

Kubota Y, Petras RE, Easley KA, Bauer TW, Tubbs RR and Fazio VW (1992) Ki-67-determined growth fraction versus standard staging and grading parameters in colorectal carcinoma. A multivariate analysis. *Cancer* **70**: 2602–2609

Lehy T, Abitbol JL and Mignon M (1984) Influence de la preparation rectale par lavement sur la proliferation cellulaire dans la muqueuse rectale normale de l'homme. *Gastroenterol Clin Biol* **8**: 216–221

Lindmark G, Glimelius B, Pahlman L and Enblad P (1991) Heterogeneity in ploidy and S-phase fraction in colorectal adenocarcinomas. *Int J Colorectal Dis* **6**: 115–120

Mulder AH, Van Hootegem JC, Sylvester R, Ten Kate FJ, Kurth KH, Ooms EC and Van der Kwast TH (1992) Prognostic factors in bladder carcinoma: histologic parameters and expression of a cell cycle-related nuclear antigen (Ki-67). *J Pathol* **166**: 37–43

Mullan FJ, Wilson HK, Majury CW, Mills JO, Cromie AJ, Campbell GR and McKelvey ST (1990) Bile acids and the increased risk of colorectal tumours after truncal vagotomy. *Br J Surg* **77**: 1085–1090

Neoptolemos JP, Oates GD, Newbold KM, Robson AM, McConkey C and Powell J (1995) Cyclin/proliferation cell nuclear antigen immunohistochemistry does not improve the prognostic power of Dukes' or Jass' classifications for colorectal cancer. *Br J Surg* **82**: 184–187

Nichols GE, Frierson JR HF, Boyd JC and Hanigan MH (1996) Automated immunohistochemical assay for estrogen receptor status in breast cancer using monoclonal antibody CC4–5 on the Ventana ES. *Am J Clin Pathol* **106**: 332–338

Palmqvist R, Öberg Å, Bergström C, Rutegård J, Zackrisson B and Stenling R (1998) Systematic heterogeneity and prognostic significance of cell proliferation in colorectal cancer. *Br J Cancer* **77**: 917–925

Paradiso A, Rabinovich M, Vallejo C, Machiavelli M, Romero A, Perez J, Lacava J, Cuevas MA, Rodriquez R, Leone B, Sapia MG, Simone G and Delena M (1996) p53 and PCNA expression in advanced colorectal cancer – response to chemotherapy and long-term prognosis. *Int J Cancer* **69**: 437–441

Pietra N, Sarli L, Sansebastiano G, Jotti GS and Peracchia A (1996) Prognostic value of ploidy, cell proliferation kinetics, and conventional clinicopathologic criteria in patients with colorectal carcinoma: a prospective study. *Dis Colon Rectum* **39**: 494–503

Porter-Jordan K and Lippman ME (1994) Overview of the biologic markers of breast cancer. *Hematol Oncol Clin North Am* **8**: 73–100

Quirke P, Dyson JE, Dixon MF, Bird CC and Joslin CA (1985) Heterogeneity of colorectal adenocarcinomas evaluated by flow cytometry and histopathology. *Br J Cancer* **51**: 99–106

Ramires M, David L, Leitao D, Seixas M, Sansonetty F and Sobrinhosimoes M (1997) Ki67 labelling index in gastric carcinomas – an immunohistochemical study using double staining for the evaluation of the proliferative activity of diffuse-type carcinomas. *J Pathol* **182**: 62–67

Rew DA (1993) Cell proliferation, tumour growth and clinical outcome: gains and losses in intestinal cancer. *Ann R Coll Surg England* **75**: 397–404

Roncucci L, Pedroni M, Scalmati A, Bormioli ML, Sassatelli R, Fante R, Losi L, Di Gregorio C, Petocchi B and Ponz De Leon M (1992) Cell kinetics evaluation of colorectal tumors after in vivo administration of bromodeoxyuridine. *Int J Cancer* **52**: 856–861

Shepherd NA, Richman PI and England J (1988) Ki-67 derived proliferative activity in colorectal adenocarcinoma with prognostic correlations. *J Pathol* **155**: 213–219

Taniyama K, Suzuki H, Matsumoto M, Hakamada K, Toyama K and Tahara E (1993) Relationships between nodal status and cell kinetics, DNA ploidy pattern and histopathology of the deeply infiltrating sites in colorectal adenocarcinoma. *Acta Pathol Jpn* **43**: 590–596

Taniyama K, Sasaki N, Wada S, Sasaki M, Miyoshi N, Nakai H, Kodama S, Nakatsuka H and Tahara E (1996) Comparison of proliferative activities and metastases between two subtypes classified at the deeply infiltrating sites of colorectal moderately differentiated adenocarcinomas. *Pathol Int* **46**: 195–203

Vermeulen PB, Verhoeven D, Hubens G, Van Marck E, Goovaerts G, Huyghe M, De Bruijn EA, Van Oosterom AT and Dirix LY (1995) Microvessel density, endothelial cell proliferation and tumour cell proliferation in human colorectal adenocarcinomas. *Ann Oncol* **6**: 59–64

Weibel ER (1979) Stereological Methods. Vol 1. Practical methods for biological morphometry. Academic Press: London

Witzig TE, Loprinzi CL, Gonchoroff NJ, Reiman HM, Cha SS, Wieand HS, Katzmann JA, Paulsen JK and Moertel CG (1991) DNA ploidy and cell kinetic measurements as predictors of recurrence and survival in stages B2 and C colorectal adenocarcinoma. *Cancer* **68**: 879–888

Zarbo RJ, Nakhleh RE, Brown RD, Kubus JJ, Ma CK, Mackowiak P (1997) Prognostic significance of DNA ploidy and proliferation in 309 colorectal carcinomas as determined by two-color multiparametric DNA flow cytometry. *Cancer* **79**: 2073–2086

*© Cancer Research Campaign 1999*
